# Analysis of the Effects of Neutron Radiation on Cellulose Linen Fabrics Using Non-Destructive Testing

**DOI:** 10.3390/polym16233401

**Published:** 2024-12-03

**Authors:** César Barta, María Fernández-Álvarez, Elisa María Ruiz-Navas

**Affiliations:** 1 Departamento de Ciencia e Ingeniería de Materiales e Ingeniería Química, Universdad Carlos III de Madrid, Avenida de la Universidad, 30, 28911 Leganés, Spain; emruiz@ing.uc3m.es; 2 Electroceramic Department, Instituto de Cerámica y Vidrio (ICV), CSIC, Kelsen 5, 28049 Madrid, Spain; maria.fernandez@icv.csic.es

**Keywords:** fluorescence, Raman spectroscopy, ATR-FTIR, neutron irradiation, cellulose, linen, cultural heritage

## Abstract

This work describes the effects of using neutron irradiation on cellulose and non-destructive methods to analyze linen fabrics of high heritage value. For this purpose, 8 samples were irradiated with increasing doses of neutrons and gamma rays up to 166 kGy of total dose. The samples were characterized by techniques such as ultraviolet luminescence, attenuated total reflectance Fourier transform infrared (ATR-FTIR) spectroscopy, Raman spectroscopy, and the nuclear magnetic resonance (NMR) technique. The luminescence of linen fabrics in the ultraviolet range increases markedly with dosage. Some chemical changes were also perceived by the ATR-FTIR spectra in the linen samples. Similarly, the fluorescence background observed in Raman spectroscopy intensifies with dosage. Due to the heterogeneity of the textile cellulose, NMR offers limited applicability for detecting neutron doses in cultural heritage fabrics. Radiation is applied for preservation against microorganisms in cultural heritage objects where the damage is to be assessed. This radiation can occasionally be found after earthquakes or in volcanic archaeological sites, which could question its dating using carbon 14. Despite some limitations encountered due to working with commonly used linen fabrics, the techniques employed in this study have made it possible to observe trends between the responses obtained and the irradiation dose for each linen sample.

## 1. Introduction

Tests on irradiated cellulosic samples began in the middle of the last century, informing several articles that were not always in English. Many of these studies dealt with ionizing radiation, usually gamma radiation, for which the mechanism for modifying cellulose has been analyzed [1]. The irradiation of cellulose, specifically with neutrons, was then documented [2]. Although there is a modern study from 2019 detailing the effects of neutron irradiation on polymers [3], it does not include cellulose, even though it is the most abundant biopolymer on Earth [1].

The effects of irradiation with pure thermal neutrons at the same dose in terms of *n*/cm^2^ are much smaller than in the case of irradiation with the full energy range of neutrons, together with gamma rays [4]. For fast neutrons, the three basic elements of cellulose (H, C, and O) behave similarly. For thermal neutrons, only hydrogen counts. Chemical changes occur mainly with fast neutrons and gamma rays, not with thermal neutrons. Each fast neutron interaction deposits about 10 times more energy than the absorption of a thermal neutron [5].

In the case of fast neutrons, 60% of the absorbed energy is taken up by hydrogen. The fast neutron beam collides elastically with the atomic nucleus, scattering the neutrons and ejecting the nucleus (a proton). The proton interacts with the electrons of other atoms, causing the excitation and ionization of the surrounding molecules [5] in a radius of ≤500 nm [6]. The equivalent effect of ionizing radiation, such as gamma rays, to the effect of non-ionizing radiation, such as neutrons, can be explained by the recoil protons generated by neutron irradiation, since protons are ionizing particles.

Regarding gamma rays, the Compton effect occurs, resulting from interactions with the electrons of atoms, usually from the outer shell, causing further excitation and ionization, which leads to chemical changes. The secondary protons from fast neutron interactions, along with Compton-effect electrons from gamma rays, attack the glycosidic bonds (C-O-C) on the backbone of cellulose chains [1], which require about 9 eV to break [7].

In the case of thermal neutrons and based on the known cross-sections, the main interaction is scattering, which accounts for more than 99% of thermal neutron interactions [8]. The low energy of the thermal neutron allows it to shake the molecular chain, but not to break it [5]. In addition, the other interaction is absorption, which leads to the transmutation of the present elements [9]. Almost all the absorption occurs with hydrogen and, as a result, deuterium is formed, emitting a gamma ray of 2.2 MeV. In a much smaller proportion, the formation of radioactive isotope carbon 14 also occurs, originating from ^13^C, ^17^O, and nitrogen impurities. In this last case, a proton with 0.58 MeV [10] is emitted as well. Protons typically deposit nearly all their energy into the sample, whereas gamma rays can escape, releasing only a small percentage of their energy into the sample [5]. In absorption by isotope oxygen 17, ^17^O, an alpha particle is also emitted; although the incidence of absorption is small, it has a secondary ionizing effect.

Despite some studies [11] that found that the effects of gamma rays are similar to those of neutron radiation, in fact, neutron absorption never happens in the case of gamma rays. The effects of pure neutron irradiation have already been studied but, in our case, the samples received both types of radiation: gamma and neutron. Other studies [12] found that the effect produced in the case of neutrons was twofold and that gamma radiation only produced the effect of oxidation, while neutrons produced hydrolysis too.

A concomitantly high fluence of the three types of radiation (thermal neutron, fast neutron, and gamma), as in this study case, could produce unexpected effects. For this reason, the importance of the rate dose considered in previous works is reviewed here. The impact of an intense dose rate is analyzed and the origin of the observed “step” (change of trend) in some parameters when passing from a low dose to an intermediate one requires further investigation.

The molecular changes that produce the purportedly observed ultraviolet (UV) fluorescence effect [13] should also be identified with the help of other methods.

Of the various cellulose fibers, cotton is the fiber for which changes in properties due to radioactive radiation have been studied in the greatest detail [2,4,11,14,15]. Recent reports show the results, mainly of gamma rays on cotton, up to doses that are an order of magnitude higher than the maximum dose in our study [16]. Cotton and linen are very similar in their chemical composition and some detection methods such as infrared spectroscopy (FTIR) are not able to differentiate them from each other [17]. Therefore, in this work, linen (flax) has been selected as a fabric with behavior against radiation that is quite similar to that of cotton but that has more archaeological interest because of the earlier use of linen in human history. Besides, due to that similitude, some of the results of this work could also be applied to cotton and other natural cellulosic fibers.

Finally, it has to be pointed out that previous research used destructive and non-destructive methods to elucidate the properties of cellulose. In this work, non-destructive characterization techniques were selected since they could be applied to cultural heritage clothes of great value. In these cases, the less invasive the method is, the better. UV and Raman spectroscopy work without contact and do not need the removal of any of the sample. They can be applied in situ with ATR-FTIR. Testing the mechanical properties was out of the scope of this study. The results of this work can be used to assess damage, if and when irradiation is applied for conservation and preservation against microorganisms [18], and to address possible inaccuracy in the radiocarbon dating of archaeological fabrics found in volcanic and seismic sites [19]. The effects of irradiation are also used for forensic investigation [20]. It could also be applied to elucidate age in the case of some controversial dating cloths [21]. Neutrons are also present in the aerospace industry. In each of these scientific fields, it will be useful to improve the knowledge of changes induced by irradiation, including neutrons. Although the main value of cultural heritage goods is intangible, their loss or damage can lead to a decrease in tourism and a negative impact on local economies [22]. The forensic and aerospace fields also move huge amounts of money [23]. A procedure to determine whether a linen cloth was subjected to neutron irradiation and to estimate the dose received would allow the analysis of damage after nuclear weapon tests or radiological accidents. If providing an absolute value is an ambitious challenge, at least the possibility of showing a gradient in fabric is more realistic.

## 2. Materials and Methods

### 2.1. Linen Samples

The linen used in testing was purchased from Rawganique Inc., Blaine, WA, USA and it is made from 100% organically grown European flax. The linen is chemical-free, unbleached, and undyed, and it was woven in-house at Rawganique Atelier in Europe to ensure true purity. The typical composition of linen (flax) is 75% cellulose, 14% hemicellulose, and 3% lignin, with wax and other unidentified components making up the remainder of the composition [24,25]. The woven texture of the fabric is simplest in taffeta. For the neutron irradiation process, the linen was cut into pieces of approximately 11.4 × 7.0 cm.

### 2.2. Samples Irradiation

The samples were irradiated with a neutron flux reactor at the University of Massachusetts Lowell Research Reactor (USA). The reactor used was the in-core sample facility Basket D2, which is capable of producing a thermal neutron flux. The samples were prepared by placing the rolled linen in semi-clear polypropylene cylindrical vials 7 cm high and 2.5 cm in diameter. Eight irradiated samples, 1 to 8, as shown in Table 1, were used for the analysis. The doses (*n*/cm^2^) applied ranged from 2.5 × 10^14^ to 1.0 × 10^16^ with a relative error of 0.18%, according to the facility specifications. The equivalent dose in kGy for neutrons and gamma rays is given below, as well as the total dose. The control sample (Table 1) is the original linen without irradiation.

To accumulate the doses, a specific neutron fluence rate was combined with the required time, as indicated in Table 1. The maximum irradiation time was 16 min and 40 s. During the irradiation, the temperature remained between 26.7 °C and 32.2 °C, set by the cooling water flow. These conditions exclude any additional heating effects. The spectral energy of the neutron radiation was roughly distributed as 63% thermal neutrons, 19% epithermal neutrons, and 18% fast neutrons, according to the characteristics of the reactor. In addition to the energy deposited by the neutrons, there was also a substantial and nearly equivalent dose of gamma rays, which could also affect the samples.

### 2.3. Ultraviolet Fluorescence Setup

Purported fluorescence intensity was previously measured from the irradiated linen samples. The setup used to take UV photos and the description of the test was described in a previous work [13]. We detail herein the spectral characteristics of the components used. The UV source used was a Convoy S2 model equipped with a 2-millimeter-thick Hoya U-340 bandpass filter (Taipei City, Taiwan). It used an LG 365 nm LED (model-LEUVA33U70RL00), which produces a sharp peak of UV light centered at 365 nm and ranging between 340 and 400 nm. The 2 mm Hoya U-340 filter effectively cuts off UV light at and above 400 nm.

The camera used to capture the photos was a Canon EOS Rebel T7 (Canon Inc., Taichung, Taiwan), equipped with a Tiffen 52 mm Haze-2A (Tiffen Company, New York, NY, USA) visible and infrared long pass filter. The intense circular region generated by the UV flashlight in the center of the sample was used for the analysis.

A new preliminary analysis of true fluorescence was carried out with the FS5 fluorescence spectrometer (Edinburgh Instruments, Edinburgh, UK) at 365 nm, and the fluorescence emission was recorded by scanning the emitted wavelength in the visible range (500 to 700 nm).

### 2.4. ATR-FTIR

Reflectance Fourier transform infrared spectroscopy (ATR-FTIR) has been demonstrated to be a suitable, non-invasive option for the chemical characterization of textile fibers [17]. To analyze the irradiated and non-irradiated samples in this study by FTIR, a Cary 630 spectrometer from Agilent Technologies with a diamond ATR configuration was used. A “blank” background spectrum was collected immediately before every sample was scanned. For each sample, six spectra were recorded for different spots on each side of the sample. To reduce the distortion of the non-uniformity of the fabric, a large number of spectra were taken at different points. Each spectrum was the average of 120 scans in the range of 4000–650 cm^−1^, with a spectral resolution of 4 cm^−1^. The Happ–Genzel apodization mode and Mertz phase correction were selected for this study. The final average spectrum of each sample was calculated from twelve acquisitions. We applied the same baseline subtraction algorithm to every sample.

To analyze the effects of low and intermediate neutron doses, the spectrum was divided into bands that can be ascribed to several functional groups (Table 2), and the areas under these bands were calculated.

The distribution of the spectrum in these bands tries to take into account the identified peaks most likely to be affected by the radiation [1,14,27,28]. Among these bands, the one at 1136–800 cm^−1^ showed the lowest variability in terms of standard deviation over the average among the range of doses. Therefore, the spectra were normalized to this band at 1136–800 cm^−1^ to show the dependence of the vibrational groups on the dose. The peak at 1060 cm^−1^, which falls within the band at 1136–800 cm^−1^, was also used to normalize the FTIR spectra in previous works [6,29]. The dependence of ratios on the dose was studied using the ratio of the areas under the different peaks of the processed spectra. The shift in the maximum intensity of the peaks can also refer to certain phenomena that are involved [1].

In particular, this type of analysis tries to find the impact of the neutron dose on the crystallinity, considering the following indicators of crystallinity [30,31]:

Ratio between 1372/2892 cm^−1^ (total crystallinity index, TCI), (1)

Ratio between 1429/893 cm^−1^ (lateral order index, LOI) (2)

and

Ratio between 3336/1336 cm^−1^ (hydrogen bond intensity, HBI). (3)

**Figure 1:**
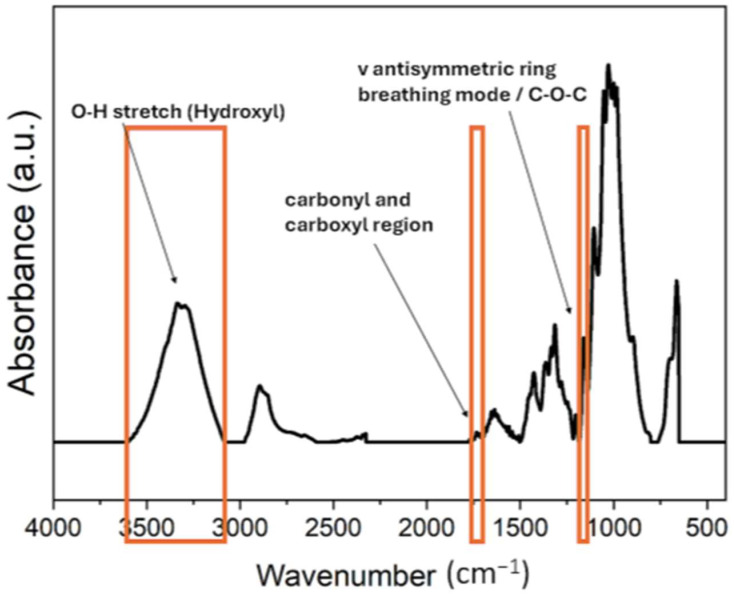
ATR-FTIR spectrum of linen, with the selected bands indicated.

The values in Table 2 were used to estimate these indexes. An analysis was also performed on the following bands (Figure 1):

3600–3070 cm^−1^, O-H stretch (hydroxyl)

1700–1760 cm^−1^, carbonyl and carboxyl region

1136–1180 cm^−1^, β-glycosidic bonds, C-O-C.

### 2.5. Raman Spectroscopy

The samples were analyzed by Raman spectroscopy, using a non-confocal portable B&W Tek iRaman device (B&W Tek, Newark, NJ, USA), coupled to a microscope with a 20× objective and a 785 nm laser. To reduce the potential damage to the samples, the power of the laser was reduced as much as possible while still maintaining a sufficient signal-to-noise ratio. All Raman measurements were performed with a laser power of 44.6 mW, an integration time of 3 s, and five accumulations in each measurement. Twelve measurements were performed on each sample, obtaining the average Raman spectrum of each sample.

### 2.6. Nuclear Magnetic Resonance

The most useful nucleus for detecting possible modifications to linen fibers due to radiation with the nuclear magnetic resonance (NMR) method is ^13^C. The ^13^C NMR spectrum allows for the estimation of the ratio between the crystalline and amorphous phases of the cellulose, since the crystalline phase corresponds to the band from 86 to 92 ppm, and the amorphous phase corresponds to the band from 80 to 86 ppm [1].

The measurements were carried out with a Bruker 400 Avance Neo NMR device (Bruker, Billerica, MA, USA), consisting of a superconducting magnet with an 89 mm Ø aperture, operating at 9.4 T (Larmor frequencies of H-1: 400.14 MHz and C-13: 100.61 MHz). The solid-state ^13^C measurements were carried out with a standard Bruker 4 mm probe, CP (cross-polarization)/MAS (magic angle spinning), and double resonance. The rolled-up linen samples were introduced into ZrO_2_ rotors with 4 mm Ø. The C-13 spectra were acquired using a pulse sequence with cross-polarization and magic angle rotation. Specifically, a contact time of 2 ms, a repetition time of 3 s, a rotation speed of 8.5 kHz, a decoupling of 62.5 kHz, and 8000 scans were averaged. A 5 Hz filter was used for processing. A strip of approximately 15 × 25 mm^2^, taken from the control sample, and eight strips of the same size from each of the irradiated samples were tested with NMR for C13 chemical shift changes. The samples’ weights ranged from 61.5 to 68.4 mg.

The analysis of the results was performed by comparing the normalized area (concerning the analyzed sample weight) of the C-13 signal at 89.2 ppm (crystalline phase of carbon 4) of irradiated samples, with the normalized area of the same signal in the ^13^C spectrum corresponding to the control sample.

## 3. Results and Discussion

As explained above, the irradiation to which the samples were subjected involved neutrons and gamma rays. Neutrons deposited about 48% of the total energy, while gamma rays contributed the remaining 52%. Among the neutrons, about 63% were thermal and the other 37% were fast neutrons. The effects (deposited energy) of the thermal neutrons are less than 1/10 of the energy deposited by fast neutrons [5].

For the irradiation process, the flux intensity (dose rate) for samples 5 to 8 was increased by a factor of 10 compared to samples 1 to 4, to achieve high doses in a short time. It is necessary to understand the dynamic of the chemical change processes. To suggest a hypothesis about the process causing the observed effect, this dynamic must be thoroughly analyzed, as follows. The interactions between ionizing radiation and organic material occur in the following three-step process [32].

In the case of gamma rays, the initial process is ionization by displacing an electron, usually from the external shell in a covalent bond. When an electron is extracted from its ground state, a positive ion is created. According to List et al. [1], this interaction is the first of a three-step sequence. The ionization potential for electrons in most organic molecules is in the range of 10 to 15 eV [1]. Gamma radiation can produce numerous ionizations, due to its energy content. This is followed by the formation of free radicals, eventually leading to alterations in the molecular structure.

For neutron interactions, the initial effect comes from the displacement of protons (the nucleus of hydrogen atoms) or absorption reactions. Consequently, electrons are ejected from their fundamental configuration, causing ionization as a secondary effect following the initial interaction. Neutron irradiation produces effects like those of gamma radiation but involves additional steps.

Free radical formation occurs due to previous ionization. The excited electrons recombine with positively charged ions that were also created by the previous ionization, producing excited functional groups. These groups break apart into free radicals that initiate more reactions [9]. These free radicals can be produced by cleavage of the hydrogen atom and cleavage of the OH group [33]. Ions and free radicals that form after cellulose irradiation continue to evolve with time in the range of hours, days, and even months, before becoming trapped and ceasing to move [34]. Chain scission, which reduces the degree of polymerization, can continue for days [5].

Finally, modifications and changes to the molecular structure are established. Typical effects include crosslinking, polymerization, and chain scission. Crosslinking forms transverse covalent bonds between the polymer chains, while scission breaks the chemical bonds in these chains [35]. The crosslinking increases the strength while the chain scission weakens it [2,36]. Crosslinking could play a key role in the samples studied in this work because it can appear in the early stages of the irradiation process. This effect is more common in the amorphous regions of the cellulose [37]. In contrast, chain scission involves breaking the chemical bonds in the cellulose chains. Breaking the chain requires around 9 eV [7], which reduces the chain’s length and decreases its degree of polymerization (DP). DP measures the length of the chain or the number of monomers in it. Crosslinking does not prevent chain scission, so both processes can occur simultaneously [4]. An analysis of cellulose behavior after irradiation should take into account the combined effects of crosslinking between nearby chains and chain scission [35].

Chain scission results in the production of carbonyl (C=O) and carboxyl (-COOH) groups [1], which are not present in the pristine chemical structure of cellulose. However, Blouin et al. [15] demonstrated that the formation of these groups, as well as chain cleavage, is minimal at gamma radiation doses below 44 kGy. This dose corresponds approximately to sample 5, considering the total dose. Only samples 6 to 8 in this study accumulated this dose or higher. The observations by Blouin are only indicative in our case because he applied gamma rays, and there are differences in the formation of functional groups when thermal neutrons are used for irradiation, as in this work [15].

### 3.1. Ultraviolet Fluorescence Study

**Figure 2:**
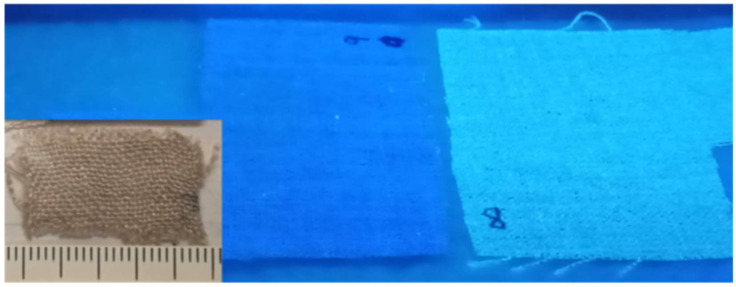
Total visible intensity under a Wood light: left, control sample; right, sample 8. Left corner inset: Close-up of part of the sample under natural light.

Under UV illumination (laboratory lamp), there was an evident difference between the control non-irradiated linen sample (left, Figure 2) and those that had been irradiated (right, Figure 2). This observation was interpreted in a previous work [13] as an increase in fluorescence intensity in the irradiated samples. However, the mechanism responsible for this change was not explored further. This study attempts to understand the cellulose changes that account for that effect.

**Figure 3:**
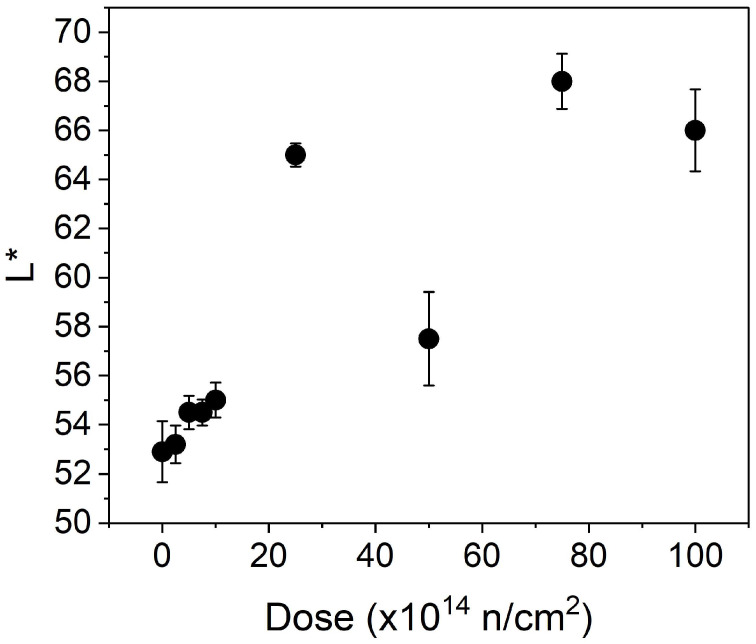
L* values of all samples. Processed from [13], with permission from the MacAvoy © Science publishing group.

The visible fluorescence interpretation came from an analysis of photographic images taken with a camera provided with filters. Figure 3 shows the results for the mean total intensity, L*, of the center of the neutron-irradiated and non-irradiated linen. This L* is the image intensity of the CIE L*a*b color space, where color is contained in the a–b plane. The CIE space is another representation of the typical RGB (red, green, and blue) description of light (see Ref. [13]). When analyzing data, it can be seen that the total intensity shows an increasing trend along with the dose. The Tiffen Haze-2A filter blocks UV light and allows the light to pass through, with a cutoff wavelength of approximately 400 nm. The Tiffen filter transmits approximately 50% of the light at 418 nm. In Figure 3, a “step” in total visible intensity is observed between sample 4 (1 × 10^15^ *n*/cm^2^) and sample 5 (2.5 × 10^15^ *n*/cm^2^). This step concurs with an increase in the dose rate that passes from 10^12^ to 10^13^ *n*/cm^2^/s. However, the UV fluorescence of sample 6 exhibited a different trend compared to samples 5, 7, and 8. This reduction in fluorescence was verified and repeated on several occasions. It is not a problem with the fluorescence set-up conditions. The greatest variance, associated with sample 6, leads one to think that the inhomogeneity of the original fabric, such as natural impurities, could be an explanation. An inaccurate irradiation setup is also possible, even though the other characterization techniques, as discussed in the following sections of this article, did not detect such an anomaly. Therefore, no more specific conclusions can be drawn.

According to the literature [38,39,40,41], cellulosic linen shows an emission peak around 450 nm when the UV excitation is around 365 nm, which is the central wavelength of the UV lamp in Ref. [13]. Some of the chromophores attributed to this fluorescence are caffeic acid, lignin, phenolic compounds, flavins, and alkaloids [42]. The apparent color of the control sample corresponds well to 450 nm because this is the perception of the human eye for violet-blue wavelengths. The setup of Ref. [13] allows for recording this band. However, the irradiated sample does not show this color; instead, it shows one that is more centered in the visible spectrum.

In addition to the observed visual color and the literature review, a preliminary spectroscopy test of the neutron-irradiated sample led to suspect the loss of the original fluorescence at 450 nm, even at low doses. Another possible autofluorescence of cellulosic materials that appears at about 570 nm when excited at 522 nm [43] may be involved in the luminescence observed in the irradiated samples. According to that reference, an explanation for the autofluorescence is the possibility that glycosidic bonds (C-O-C) in the cellulose randomly form unsaturated bonds. After neutron irradiation, the glycosidic bonds probably decrease, and the carboxyl groups (–COOH) increase (see the ATR-FTIR study herein, detailed below). This would indicate a possible reduction of autofluorescence at 570 nm for the irradiated samples in this study.

However, funerary linen fabrics were found to emit greater fluorescence intensity than modern linen in almost all the wavelengths of the spectrum from 300 to 700 nm [42]. Laude [44] also documented an increase in luminescence at 550 nm when a proton-irradiated linen was excited at 473 nm.

The pristine linen sample appears blue in the photographic image taken under UV light (Figure 2), while the irradiated sample appears brilliant white. Considering the reduction of fluorescence in the irradiated samples, a possible contribution by the light reflection and scattering effect should be considered.

A radiation incident (I_α_), as in the UV illumination (Figure 2), upon a fabric sample is either scattered (I_s_), absorbed (I_A_), or transmitted (I_T_) [45]. Transmission occurs through openings between the threads (I_TO_) or is transmitted through the fibers (I_TF_).

I_α_ = I_s_ + I_A_ + I_T_ (4)

I_T_ = I_TO_ + I_TF_ (5)

I_α_ = Radiation incident upon a fabric

I_s_ = Radiation scattered

I_A_ = Radiation absorbed

I_T_ = Total radiation transmitted

I_TO_ = Radiation transmitted through openings between the threads

I_TF_ = Radiation transmitted through the fibers (transparency).

Neutron radiation can change the absorption and transmission through fibers. Hypothetically, neutron irradiation breaks the fluorescent bonds in the original linen, and the exciting UV light is no longer absorbed, and this light is scattered more and more as the damage from the irradiation increases with the dose. The total visible intensity of the samples found in Ref. [13] could make an important contribution to scattering, while the increase in total intensity may be related to changes in the UV light absorption, reflection, and diffraction of the irradiated material.

As a first approach to any explanation of the mechanism causing the increase in total visible intensity that was observed in our irradiated samples of linen, it could be proposed that the UV absorption at 365 nm of the original linen is canceled when it is irradiated, and the scattering is increased by the reflection and diffraction of the incident UV light.

However, this hypothesis needs to be corroborated by complementary analyses, such as thorough UV absorption and emission spectroscopy and a reflectance test, to confirm whether the increase in the total visible intensity is from fluorescence or scattering.

In addition, Figure 3 shows a substantial increase in total intensity between sample 4 and sample 5. For sample 5 and samples 6 to 8, the dose increases and the dose rate also increases. When the dose rate increases by an order of magnitude, the density of the radicals also rises, and the subsequent reactions involving these radicals may vary. Many cleavages of the hydrogen atoms in adjacent chains occur concurrently with the shaking of these chains by the thermal neutrons, leading to a greater number of nearby radicals that can link the polymer chains, particularly in the amorphous phase. As a result, the luminescence effect increases significantly.

Some studies [5] found that several properties of cellulose depend almost exclusively on the total dose, while Teszler [11] found no influence of the dose rate on the polymerization effect (DP). However, if the proposed hypothesis were true, the crosslinking could depend on the flux intensity. In fact, in a previous work [4], it was observed that the crosslinking and the chain scissions, which determine the polymerization, can be independent. The DP shows, on the other hand, a monotone decrease with an increase in dose [11,15]. This depends on the glycosidic bonds (C-O-C) between monomers. However, this effect may happen as secondary damage after the earlier damage to the hydrogen bonds [46]. With a high dose rate of neutrons and the corresponding high density of hydrogen bonds, crosslinking can predominate because the scission of the C-O-C bonds can only use the residual radicals after the first step. This possible explanation for the phenomena observed should be studied more deeply. In the same way, the previous explanation of the scattering after neutron irradiation has considered only the main components of the cellulose; the role of the impurities in linen remains a line of inquiry to be explored.

### 3.2. ATR-FTIR Characterization

**Figure 4:**
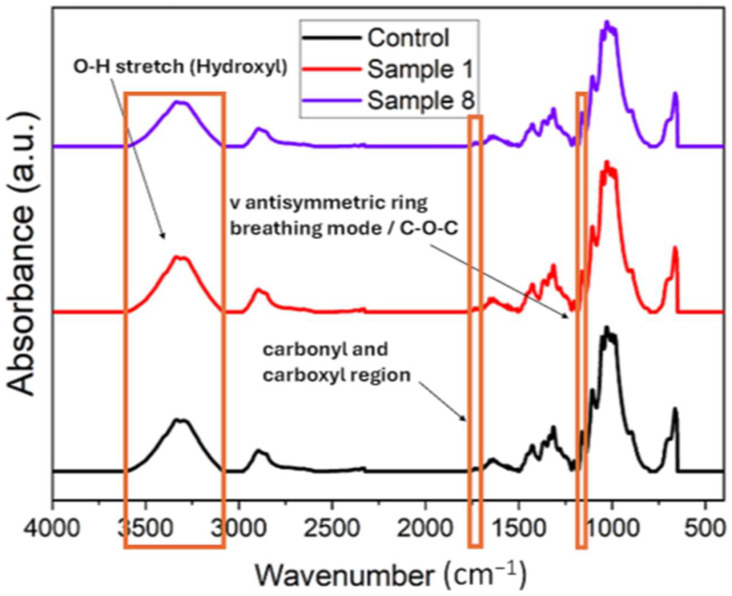
ATR-FTIR spectra of the control sample and samples 1 and 8.

The results obtained by ATR-FTIR treatment have been used to analyze the changes in the vibrational groups as the dose increases in the different samples and to understand the physicochemical changes of any parameter that could be used as an irradiation index. Figure 4 shows that there are no evident changes in the bands. The stability of the 1700–1500 cm^−1^ band, which is normally sensitive to environmental humidity, confirms that the recordings were made under equivalent environmental conditions.

**Figure 5:**
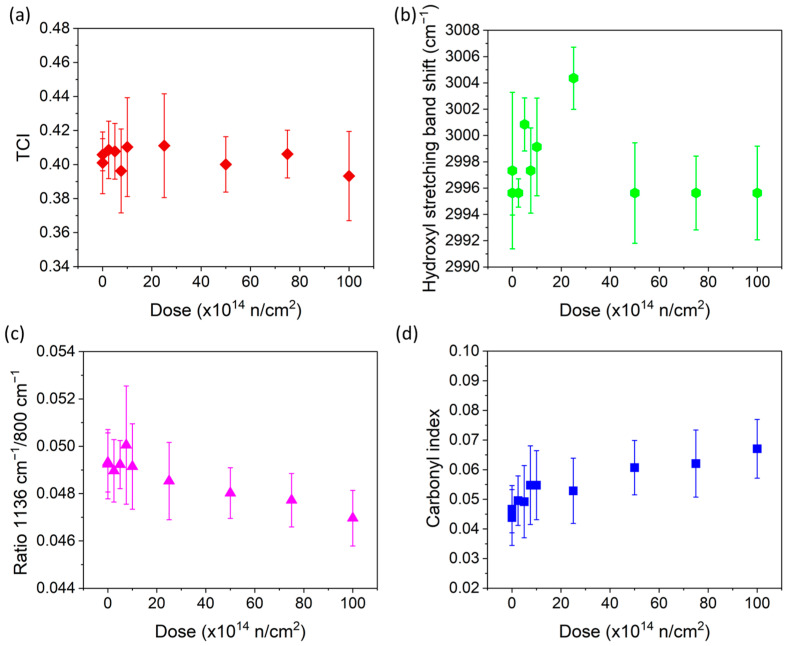
(**a**) TCI. (**b**) Hydroxyl stretching band shift (cm^−1^). (**c**) Ratio between the bands at 1180–1136 cm^−1^ (ring breathing mode/C-O-C) and 1136–800 cm^−1^. (**d**) Ratio between the bands at 1760–1700 cm^−1^ and 2980–2600 cm^−1^ (carbonyl index C=O), calculated for all samples.

To begin with, the indices of crystallinity (TCI, LOI, and HBI [30,31]) were calculated for all samples. Figure 5 shows the TCI as an example, where it can be seen that this index hardly changes and remains within the range of uncertainty for the different doses. This is also the case for the other two parameters (LOI and HBI). There is a large variability in the numerous ATR-FTIR measurements that are taken throughout a single sample, due to the nature of the linen fabric’s raw material, which makes it difficult to use ATR-FTIR to determine the crystallinity of cultural heritage fabrics.

According to the literature, the hydroxyl group is usually involved in cellulose changes and the displacement of the peak can inform these changes [1], A shift toward a higher wavenumber is observed in the low dose range (Figure 5b) in the band of the OH stretch (3600–3070 cm^−1^, hydroxyl group). Despite the large uncertainty already mentioned above, an increase in the intermolecular hydrogen bonds at the expense of intramolecular hydrogen bonds can be assumed [14]. This confirms a possible increase in the crosslinking at low doses. However, for doses higher than 2.5 × 10^15^ (sample 5), there is no change in the shift of the band (Figure 5b), which requires further analysis.

The β-glycosidic bonds of C-O-C are necessary to keep long chains and, thus, polymerization. According to the literature [5,11,15], the polymerization decreases with the dose, even at low doses. The band at around 1160 cm^−1^ (1180–1136 cm^−1^) is associated with the C-O-C bonds and, in fact, it shows the expected decrease with the dose, although it is associated with enough uncertainty (Figure 5c).

In contrast, the carbonyl and carboxyl groups should appear as an effect of the radiation, as explained above. The bands in the 1760–1700 cm^−1^ range account for the presence of the carbonyl group, which is produced by the oxidation of the OH group [47]. The ratio between 1714 and 2927 cm^−1^ is a carbonyl index [48,49]. The ATR-FTIR obtained from the samples confirms this tendency (Figure 5d).

According to the objective of this work regarding the challenge of finding a gradient of neutron irradiation on a large piece of fabric, these two ratios (1180–1136 cm^−1^/1136–800 cm^−1^ and 1760–1700 cm^−1^/2980–2600 cm^−1^) could offer good possibilities. Therefore, as a result of exploring different coefficients for the linear algorithm, a specific linear combination of the stated ratios with the coefficient −9.5 is proposed, to find a correlation between the neutron dose and some non-destructive ATR-FTIR indicator, as follows:

(6) $$I_{n}=(\frac{\int_{1700}^{1760}Idf}{\int_{2600}^{2980}Idf}-9.5\cdot \frac{\int_{1136}^{1180}Idf}{\int_{800}^{1136}Idf})\cdot 100$$

(7) $$I_{n}=0.00416\cdot dose-42.017$$

where *I_n_* is the *neutron* irradiation *index* as a combination of the 1710 cm^−1^ and 1160 cm^−1^ bands, as a function of the *dose*.

**Figure 6:**
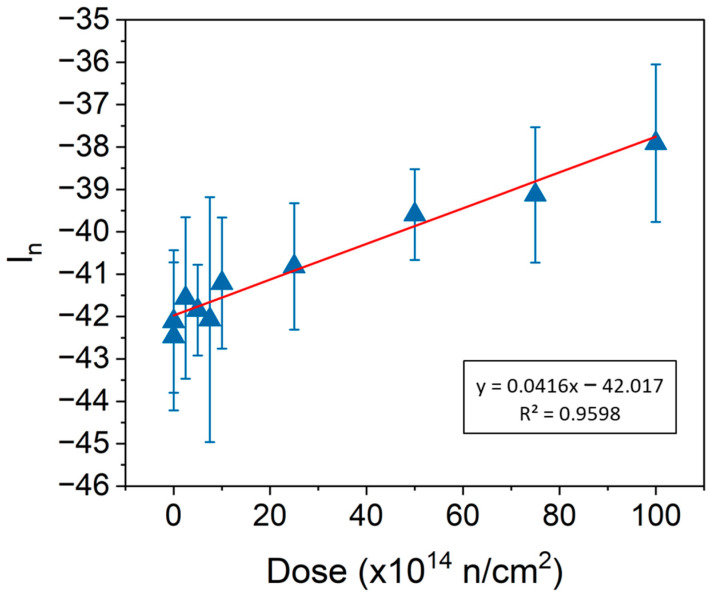
The *In* combination of bands 1710 cm^−1^ and 1160 cm^−1^ as a function of the dose (blue triangles). The red line is the linear regression.

Figure 6 shows the good correlation found between *I_n_* and dose. Finding and employing this indicator could allow scientists to detect the possible dose gradient of the neutron irradiation undergone by a long linen cloth. However, a significant number of measures must be performed to reduce the uncertainty. Because linen and cotton have almost the same ATR-FTIR spectrum [17], the proposed *In* index could be applied to both cotton and linen.

### 3.3. Raman Spectroscopy Characterization

Raman spectroscopy measurements were performed with a 785 nm laser on all samples to try and locate the different Raman bands that allow us to obtain information on the crystallinity of the different irradiated samples. Some authors have quantified this crystallinity and/or degradation by considering different Raman bands. For example, according to Botti et al. [50], some of the bands and their associated groups that can provide information are the ratio between the Raman bands at 1100 cm^−1^ and 1380 cm^−1^ (crystallinity) and the Raman bands at 1100 cm^−1^ (C-O-C), 2890 cm^−1^ (CH and CH_2_), and 1602 cm^−1^ (the C-C of the lignin).

**Figure 7:**
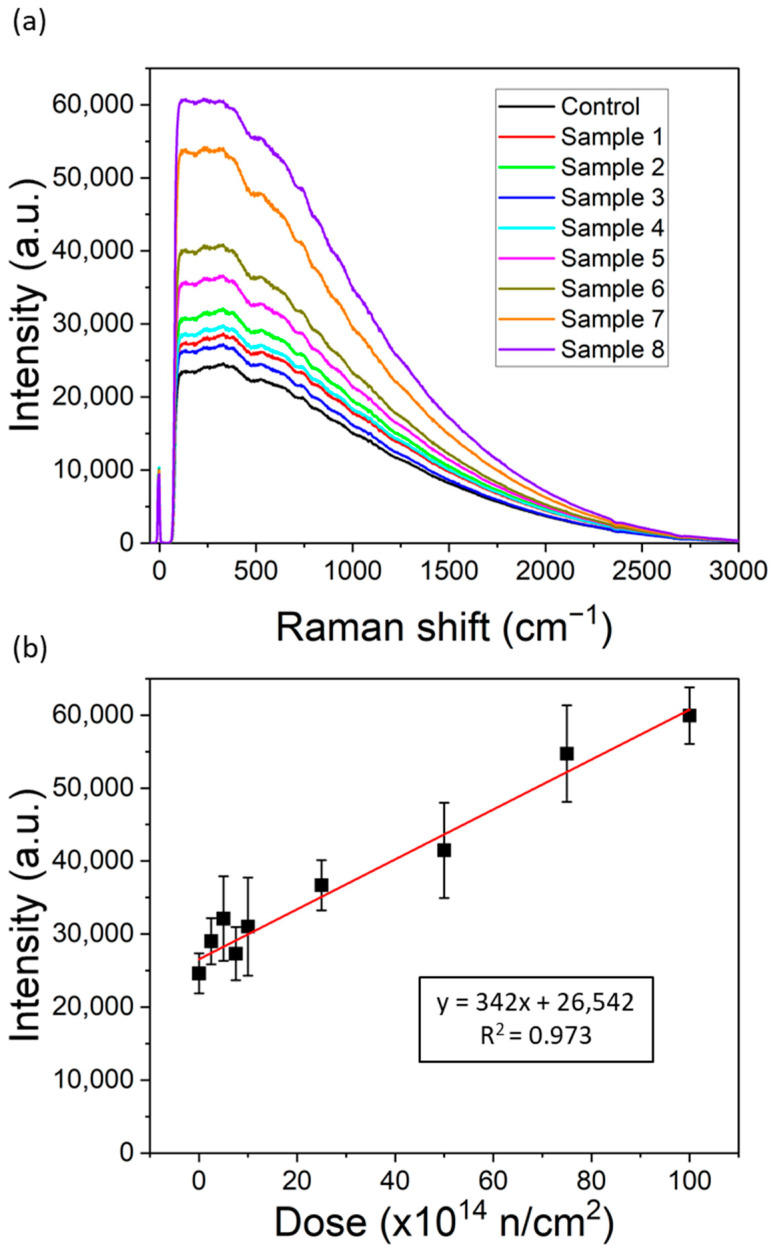
(**a**) Raman spectra and (**b**) intensity Raman quantification at 330 cm^−1^ of all samples (black squares). The red line is the linear regression.

Figure 7a shows the average Raman spectrum obtained for each linen sample. As can be seen in the figure, it is not possible to distinguish the different Raman bands that are found in the literature, since a fluorescence background predominates in all materials that hinder the Raman signal. This may be due to several reasons. Firstly, previous studies [51,52] have shown that, with visible excitation lasers, it is much more difficult to define the distinct Raman bands. This is generally attributed to the presence of a strong fluorescence background, which is typically associated with the presence of lignin in these materials [51]. To address this issue, other researchers have used FT-Raman spectroscopy with a 1064 nm laser, which allows for easier identification of the Raman bands [51].

Furthermore, it is necessary to take into account that this fluorescence background appears particularly when measured with portable equipment, since these have more basic and simple cut-off filters that do not eliminate the unwanted signal and, therefore, do not allow the Raman signal to be seen clearly [53]. Raman devices with higher resolutions allow these Raman bands to be better defined [51]. However, the use of portable equipment has great advantages, especially in high-value heritage applications, since it allows the measurements to be performed in situ, which is an important factor considered in this work. Considering the Raman spectra shown in Figure 7a, a clear trend can be observed in the fluorescence backgrounds of each sample. Samples with a higher dose of irradiation exhibit a stronger fluorescence background. The average Raman intensity at the most intense point (around 330 cm^−^¹) has been quantified in Figure 7b, along with the standard deviation, to illustrate the trends seen across the different linen samples. It can be seen that at low rates of dose, the fluorescence levels are similar, considering the deviations, but from sample 6 onward, the fluorescence background increases significantly. The results seem to have a linear trend with the dose; therefore, although the different Raman bands of the samples could not be revealed, a correlation was seen between the samples and the fluorescence background obtained by Raman spectroscopy, especially for samples given high doses. Some authors have seen this fluorescence previously in other materials, where, on some occasions, it has been attributed to the presence of lignin. Nevertheless, Raman fluorescence does not have a clear origin, as has been observed for completely different materials. It is often associated with the presence of various point defects or even radical ions, so its origin must be explained individually for each material [54].

### 3.4. NMR Characterization

**Figure 8:**
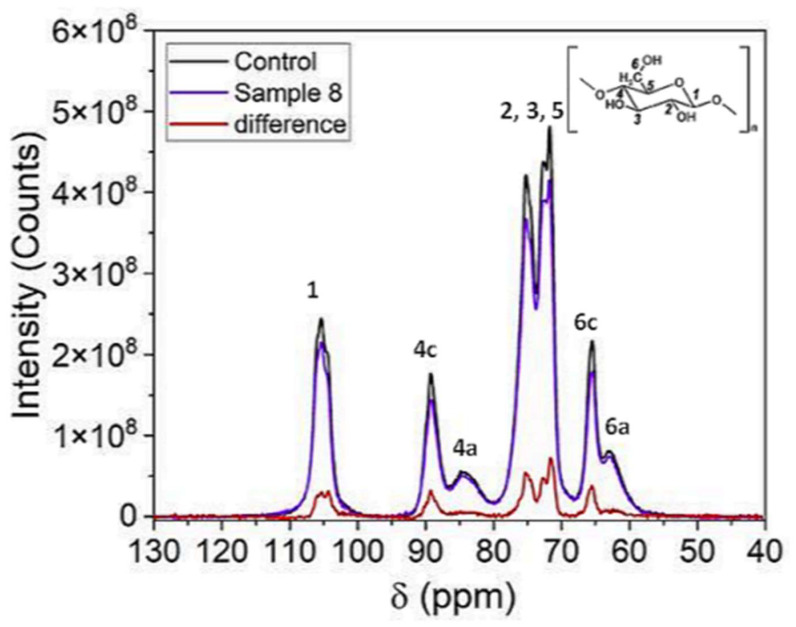
NMR spectra of the control sample and Sample 8, and the difference between both the spectra from the maximum dose sample, Sample 8, and the control sample. Numbers correspond to the indicated position in the monomer (right corner above). “c” is for crystalline. “a” is for amorphous.

The UV, FTIR, and Raman spectroscopy techniques record the surface properties, while NMR analysis characterizes the bulk of the sample. The NMR results are illustrated in Figure 8.

The spectrum shown in Figure 8 is constituted by 12 peaks. Numbers correspond to the indicated position of carbon in the monomer (right corner above in Figure 8). Peaks 4 and 6 are resolved. Those labeled “c” correspond to the crystalline phase, and those labeled “a” correspond to the amorphous phase. However, only the difference between the chemical shift (position of the peaks in the spectrum) corresponding to the crystalline and amorphous phases of the carbons in positions 4 and 6 is large enough for them to appear to be resolved in the spectrum. Peak 4c corresponds to the carbon in position 4 (Figure 8, inset drawing) of the crystalline phase, while 4a corresponds to this carbon in the amorphous phase. The corresponding codes are used for the carbon in position 6. For the carbons in the other positions, this difference is smaller, and the peaks of the two phases overlap appreciably and do not appear resolved.

**Figure 9:**
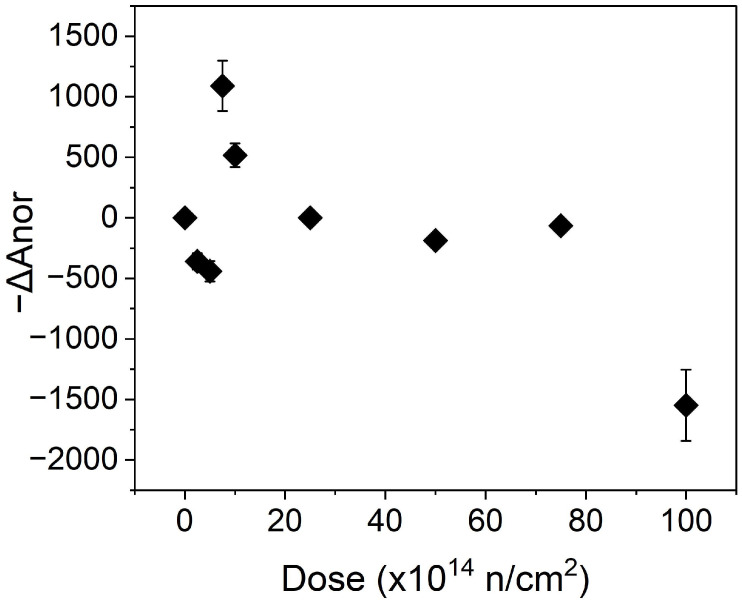
−ΔA_nor_ vs. dose, indicating the change in the degree of crystallinity for the irradiated samples with respect to the control.

As an indication of the crystalline index, the parameter ΔA_nor_ is defined as the variation of the area of the C-13 signal at 89.2 ppm/weight of the sample (normalized area), relative to the control sample. An important reduction in the crystalline index for the maximum dose applied in our study can be observed in Figure 9. For intermediate doses, the crystallinity remains almost unchanged, but for low doses, it initially shows a decrease and, subsequently, an increase compared to the non-irradiated sample. We call this unexpected evolution a “step”. The change in tendency happens between a neutron equivalent dose of 5 × 10^14^ to 7.5 × 10^14^ neutrons/cm^2^. Sasaki [5] provided results from comparable conditions when applying neutrons and gamma rays, working within similar dose ranges if we take into account the total dose. Sasaki obtained the size of the crystalline regions, which should correspond to the index of crystallinity, and observed a decrease only for doses greater than 10^6^ Roentgen (≈8.8 kGy) and a clear reduction for a dose of 2 × 10^7^ Roentgen (≈175 kGy). This decrease in crystallinity at high doses corresponds generally with our results from the NMR analysis. However, no step within the specific dose range was reported.

The experiments conducted in Refs. [55,56] also reported crystallinity at low doses and neither study shows any step within a specific range. A comparison with the results of the last reference is not fully justified, since the irradiation therein was carried out with gamma rays and an electron beam and the samples were of paper-grade bamboo pulp and microcrystalline cellulose, respectively. However, recrystallization can occur after irradiation since the short split chain can undergo recrystallization [1].

Measures of crystallinity in raw fabric seem unreliable. The results from the TCI calculated with the ATR-FTIR spectrum (Figure 5a) and from the NMR method exhibit inverse tendencies.

The results from the X-ray diffraction (XRD) tests performed by the authors but not included in this work evidenced that estimating crystallinity presents several problems [30] when the samples are fabrics made from natural linen. Moreover, it was not expected to find observable changes in crystallinity [1,57]. The heterogeneity of the cellulose crystal, due to its free growth in nature and manufacture, leads to intrinsic variability in many parameters across a single sample before being irradiated and covers variability among different irradiated samples. Therefore, XRD and NMR provide limited applicability for detecting neutron doses on cultural heritage fabrics. Moreover, they are intrusive methods because it is necessary to extract a sample from the fabric for the test, which hinders its potential application in numerous situations related to items of artistic and cultural interest.

## 4. Conclusions

Luminescence, ATR-FTIR and Raman spectroscopies, and the NMR technique, all of them non-destructive, have been employed to try and measure and define a correlation between neutron irradiation and certain cellulose properties. At least, the first three techniques can be applied in situ on cultural heritage objects. The more relevant conclusions that were drawn after carrying out this research are:

An important increase in the total light intensity has been found when illuminating fabrics in the UV range. The contribution of the thermal neutron can increase the efficiency of this effect. What was supposed to be UV fluorescence in the neutron-irradiated samples is now postulated to be light scattering.

In addition, although ATR-FTIR, when applied to cultural heritage fabrics, presents limitations when estimating crystallinity, it suggests a decrease in the band at around 1160 cm^−1^, which is associated with C-O-C bonds, and an increase in intermolecular hydrogen bonds at the expense of intramolecular hydrogen bridging when the dose is increased up to a certain level. Likewise, the increase in the carbonyl and carboxyl groups is indicated in the ATR-FTIR spectrum.

Therefore, a combination of two bands could provide an index of neutron irradiation on linen and cotton fabrics. One band is associated with the β-glycosidic bonds C-O-C (1136–1180 cm^−1^), and another is associated with the groups of carbonyl and carboxyl (1700–1760 cm^−1^). Nevertheless, the use of this technique under the conditions in which cultural objects are stored could reduce the accuracy of the results.

Raman spectroscopy also shows an increasing background fluorescence with the increase in the dose that seems to have a linear trend; therefore, although the different Raman bands of the samples could not be revealed, a correlation was seen between the samples and the fluorescence background, especially for samples receiving high doses.

Despite NMR being a non-destructive technique, this research has proven that it does not show clear utility as an irradiation indicator, due to the nature of the fabrics being examined.

Among the different techniques studied, UV luminescence and Raman spectroscopy, as non-destructive tests, could be good candidates to detect neutron irradiation. However, the identification of the structural changes that cause the total light intensity and Raman fluorescence requires more research. Further research is planned with thorough UV fluorescence spectrometry and differentiation of the effect of pure gamma rays, among other characterization methods.

## Figures and Tables

**Table 1:** Neutron irradiation conditions for the linen samples tested in this work.

Sample	Neutron Fluence Rate	Neutron Dose		γ Dose	Total Dose	Time
	*n*/cm^2^/s	*n*/cm^2^	kGy	kGy	kGy	s
1	10^12^	2.5 × 10^14^	2	2.15	4.15	250
2	10^12^	5.0 × 10^14^	4	4.30	8.30	500
3	10^12^	7.5 × 10^14^	6	6.45	12.45	750
4	10^12^	1.0 × 10^15^	8	8.60	16.60	1000
5	10^13^	2.5 × 10^15^	20	21.50	41.50	250
6	10^13^	5.0 × 10^15^	40	43.00	83.00	500
7	10^13^	7.5 × 10^15^	60	64.50	124.50	750
8	10^13^	1.0 × 10^16^	80	86.00	166.00	1000
control	-	0	0	0	0	-

**Table 2:** Assignment of the bands obtained from the ATR-FTIR spectra to the functional groups.

Wavenumber Range (cm^−1^)	Functional Group
3600–3070	O-H stretch (hydroxyl)
2980–2600	CH stretch/Asymmetric CH_2_ stretch/lignin [26]
1760–1700	C=O tensile strength carbonyl
	C=O carbonyl region/carboxyl (–COOH)
1700–1500	Absorbed water/carbonyl (C=O)
	ν(C=C) (lignin compounds)/carboxyl (–COOH)
1500–1390	δ OH primary and secondary alcohol
	δ CH_2_ scissoring motion
1390–1346	δ CH deformation
1346–1325	δ OH deformation
1325–1290	δ CH_2_ wagging
1180–1136	ν antisymmetric ring breathing mode/C-O-C
1136–1083	C-O-C ν symmetric glycosidic stretch/ring stretching mode
1136–800	CC and CO stretching; HCC and HCO bending

## Data Availability

The original contributions presented in this study are included in the article. Further inquiries can be directed to the corresponding author.
